# Repeatable Population Dynamics among Vesicular Stomatitis Virus Lineages Evolved under High Co-infection

**DOI:** 10.3389/fmicb.2016.00370

**Published:** 2016-03-31

**Authors:** Elizabeth S. C. P. Williams, Nadya M. Morales, Brian R. Wasik, Vesna Brusic, Sean P. J. Whelan, Paul E. Turner

**Affiliations:** ^1^Department of Ecology and Evolutionary Biology, Yale University, New HavenCT, USA; ^2^Department of Microbiology and Immunobiology, Harvard Medical School, BostonMA, USA

**Keywords:** cheating behavior, co-evolution, defective interfering particle, ecological dynamics, experimental evolution, RNA virus, virus ecology

## Abstract

Parasites and hosts can experience oscillatory cycles, where the densities of these interacting species dynamically fluctuate through time. Viruses with different replication strategies can also interact to produce cyclical dynamics. Frequent cellular co-infection can select for defective-interfering particles (DIPs): “cheater” viruses with shortened genomes that interfere with intracellular replication of full-length (ordinary) viruses. DIPs are positively selected when rare because they out-replicate ordinary viruses during co-infection, but DIPs are negatively selected when common because ordinary viruses become unavailable for intracellular exploitation via cheating. Here, we tested whether oscillatory dynamics of ordinary viruses were similar across independently evolved populations of vesicular stomatitis virus (VSV). Results showed identical cyclical dynamics across populations in the first 10 experimental passages, which transitioned to repeatable dampened oscillations by passage 20. Genomic analyses revealed parallel molecular substitutions across populations, particularly novel mutations that became dominant by passage 10. Our study showed that oscillatory dynamics and molecular evolution of interacting viruses were highly repeatable in VSV populations passaged under frequent co-infection. Furthermore, our data suggested that frequent co-infection with DIPs caused lowered performance of full-length viruses, by reducing their population densities by orders of magnitude compared to reproduction of ordinary viruses during strictly clonal infections.

## Introduction

Evolutionary ecology concerns interactions between biological populations and their biotic and abiotic environments. Viruses necessarily interact with host organisms (Hurst, 2000), whose metabolic functions are required for completing virus replication. However, less-appreciated aspects of virus evolutionary ecology are the relationships between individual viruses, and the role of these interactions in driving virus evolution. Viruses can indirectly affect the fitness and evolutionary fate of other viruses through interactions with the host immune system, such as cross reactivity where one infecting virus species causes the immune system to also recognize and target related viruses. In contrast, direct virus–virus interactions are possible when multiple viruses co-infect the same host cell, such as a host bacterium or an individual cell within a multi-cellular eukaryotic host. Here, one virus can directly lower the fitness of a co-infecting virus by sequestering intracellular proteins essential for virus genome replication, thus interfering with the co-infecting strain’s reproductive success (Huang and Baltimore, 1970; Horiuchi, 1983; Depolo et al., 1987; Turner and Chao, 1999; Thompson and Yin, 2010; Ojosnegros et al., 2011).

When direct ecological interactions occur, these can lead to short-term or long-term dynamics; classic examples include dynamics between parasites and their hosts (Fine and Clarkson, 1982; Potts et al., 1984) and between predators and their prey (MacLulick, 1937). These community dynamics are often driven by resource limitation and availability, principally when one community member uses another as a resource (e.g., predators consuming prey, parasites utilizing hosts) or when community members compete for the same resources. Resource competition can select for differing resource-use strategies, where a classic example is evolution of cheating versus non-cheating behavior, when resources are available in a common pool. Here, we define a cheater as possessing a mechanism (e.g., behavioral trait, molecular property) that causes it to experience a reproductive advantage relative to a non-cheater, when utilizing limited resources to create progeny. Although the evolution of cheating appears to be very prevalent in biological systems (Smith, 1982; Turner and Chao, 1999; Travisano and Velicer, 2004), the long- and short-term ecological dynamics of cheater/non-cheater interactions are seldom studied outside of mathematical theory (e.g., game theory), and are therefore poorly understood even in systems where the underlying molecular biology and genetics of cheating have been determined (Huang, 1973; Griffin and Fried, 1975; Huang and Baltimore, 1977; Lazzarini et al., 1981; Velicer et al., 1991). Do independently evolving but otherwise identical populations show equivalent dynamics when cheater genotypes evolve? Experimental evolution studies in microbes demonstrate that interaction dynamics can be highly repeatable across laboratory microcosms (Turner et al., 1996; Rainey and Travisano, 1998; Rozen et al., 2002). But microbial studies have rarely addressed the repeatability of population dynamics when virus–virus interactions, such as cheating, evolve *de novo* (e.g., Horiuchi, 1983).

To study this problem, we allowed replicate populations of viruses to evolve in the laboratory under conditions where co-infection occurred frequently. These culture conditions favored selection for viral “cheaters” defined as defective-interfering particles (DIPs) that are observed in natural virus populations, and seen in clinical samples (von Magnus, 1951; Lief and Henle, 1956; Huang and Baltimore, 1970; Huang, 1973; Huang and Baltimore, 1977; Horodyski and Holland, 1980; Taylor, 1999; Aaskov et al., 2006). Errors in genome replication can sometimes create virus mutants that have point mutations or deletions that inactivate one or more essential virus genes. However, it is important to distinguish DIPs from other types of deletion mutants, because DIPs are defined to possess two particular properties that allow them to “cheat” when co-infecting cells with ordinary (full-length) variants of the same virus. First, DIPs are ‘defective’ because these viral genotypes lack proper function in one or more essential virus genes due to a genomic deletion, which prevents a DIP from replicating in a cell unless a full-length virus is also present (Holland et al., 1991). Second, DIPs gain an advantage over otherwise identical full-length viruses during co-infection because they somehow ‘interfere’ with their replication, hence lowering the expected progeny production of ordinary viruses within the cell. These combined properties define DIPs as ‘defective-interfering’ and these traits explain why DIPs can lower the fitness (reproductive output) of full-length viruses during co-infection of the same cell. Importantly, we emphasize that an ordinary virus must be present during co-infection in order for a DIP to replicate, and this presence of the full-length virus necessarily ‘helps’ the DIP genotype. Although by definition an ordinary virus suffers reduced fitness relative to a co-infecting DIP, the rescue effect of the ordinary virus has popularized such full-length variants to be termed ‘helper virus’ in the literature (e.g., Huang and Baltimore, 1970). From an evolutionary biology standpoint, ‘helper virus’ can be considered a misnomer because it incorrectly implies that ordinary viruses are selected to beneficially interact as mutualists with DIP genotypes that arise spontaneously in the virus population. Rather, the correct interpretation is that DIPs are spontaneous mutants that parasitize ordinary viruses during co-infection, and in the remainder of the paper we avoid using the classic ‘helper virus’ terminology to avoid confusion by the reader.

Whereas genome replication errors can cause DIP mutants to spontaneously occur during most (or all) cellular infections (Huang, 1973; Lazzarini et al., 1981; Perrault, 1981), only frequent opportunities for co-infection are expected to foster persistence of DIPs. The reason is that DIPs are unlikely to persist as a pure population because they do not encode all of the functional genes necessary for completion of the virus life cycle within a cell (but see Garcia-Arriaza et al., 2004, for a rare example of multiple DIPs that coexist via complementation). In contrast, frequent co-infection allows spontaneously occurring DIP mutants to persist alongside ordinary virus genotypes, because the latter provide missing essential viral proteins and functions within the cell. Various mechanisms can account for the interference enacted by DIPs on ordinary viruses. But the smaller genome sizes of DIPs immediately afford faster rate of replication compared to full-length viruses, and this size difference alone may be sufficient to explain their competitive advantage (Huang, 1973; Lazzarini et al., 1981; Coburn and Cullen, 2002).

Regardless of the cheating mechanism, if virus co-infection is prevalent, the relative frequencies of DIPs and ordinary viruses are expected to dynamically oscillate through time (Kirkwood and Bangham, 1994). The selective advantage of spontaneously occurring DIP mutants causes them to increase in genotype frequency through time (i.e., invade when initially rare) in the viral population. As DIPs increase in frequency this forces the frequency of ordinary virus genotypes to decline, because DIPs are defined to be fitness advantaged over their full-length virus counterparts during co-infection. But when DIPs reach high frequencies in the population, most cells will be co-infected only by DIP mutants and these dead-end infections will produce no viral progeny (i.e., DIPs lack one or more essential virus genes, and two fully complementary DIP mutants have low probability of fortuitously entering the same cell). DIPs should then decrease in genotype frequency, allowing ordinary viruses to numerically recover (i.e., invade when initially rare) and the cycle repeats (Huang, 1973). Importantly, this oscillating dynamic (negative frequency-dependent selection) can be revealed by obtaining samples from the virus population through time, and enumerating only the ordinary viruses that are present in the samples. The reason is that estimates of virus densities (titers) rely on assays that place dilutions of population samples onto a substrate (e.g., cell monolayers grown in tissue culture), such that individual particles hit the substrate and give rise to progeny that locally destroy cells to create non-overlapping ‘holes’ (plaques) visible on the substrate. Necessarily, DIPs lack one or more essential genes and therefore cannot form plaques initiated by an individual particle in such plaque assays. Hence, plaque assays can reliably track densities of the ordinary virus subpopulation through time, but the DIP subpopulation remains invisible in such assays. Nevertheless, enumeration of the ordinary viruses is sufficient to prove that oscillations are occurring, and some *in vitro* experiments have classically demonstrated the expected oscillations when viruses are passaged under frequent co-infection (Huang, 1973; Palma and Huang, 1974; Horiuchi, 1983). However, these studies tended to focus on the molecular virology of DIPs rather than the repeatability of oscillatory dynamics across replicate evolved populations, which is the main goal of the current study.

Here, we employed vesicular stomatitis virus (VSV) as a model to study the repeatability of oscillatory dynamics, across virus populations that were independently passaged under frequent co-infection. VSV is a well-characterized arthropod-borne virus (arbovirus) in the family Rhabdoviridae, which naturally infects various species of insects and mammals, including occasionally humans (Letchworth et al., 1999; Rose and Whitt, 2001). VSV has a ∼11.2 kb negative sense ssRNA genome encoding five proteins: the nucleocapsid (N) protein that encapsidates the genomic RNA, phosphoprotein (P) and large (L) protein which make up the polymerase, glycoprotein (G) involved in cell-surface binding, and matrix (M) protein important both for virion formation and inhibition of host antiviral gene expression (Rose and Whitt, 2001; Lyles and Rupprecht, 2007). As with many ssRNA viruses, VSV has a high mutation rate, rapid generation time, and small genome size, making it a useful and efficient model for examining phenotypic and genotypic evolution (Moya et al., 2004). Although genetic exchange (recombination, reassortment) can be consequential for virus evolution (Perez-Losada et al., 2015), we note that recombination is either extremely rare or non-existent in VSV (Han and Worobey, 2011). VSV is generally not pathogenic to humans, and grows easily in many cell culture systems and tissues owing to its broad tropism (Hastie and Grdzelishvili, 2012). VSV cultured *in vitro* typically reaches very high titers (10^8^–10^10^ viruses per mL) within 24 h, on permissive cell types such as baby hamster kidney (BHK-21) cells. Serial passage of VSV under high multiplicity of infection (MOI; ratio of viruses to cells) allows frequent virus co-infection, conditions that promote strong selection for spontaneous DIP mutants in VSV populations (Huang, 1973; Holland, 1991; Thompson and Yin, 2010; Ojosnegros et al., 2011).

We used a single ancestral VSV strain to initiate five replicate virus populations, which were independently passaged for 20 consecutive days on BHK cells at high *MOI*. Results showed that the oscillatory dynamics of ordinary viruses were highly similar across the populations, characterized by high-amplitude oscillations between passages 0 and 10, and followed by dampened oscillations between passages 10 and 20. In addition, full-genome consensus sequencing of mixed population samples showed that parallel molecular evolution occurred across the five populations, and that most of these changes fixed early in the study. Our results suggested that negative frequency-dependent selection experienced by ordinary and DIP genotypes of VSV was highly similar across our study populations, evidenced by ordinary-virus dynamics and molecular substitutions that were repeatable and parallel across independently evolving RNA virus populations.

## Materials and Methods

### Viruses and Culture Conditions

All viruses were derived from a clonal isolate of a low-passage, laboratory-adapted strain of Mudd Summer VSV Indiana serotype that was kindly provided by E. Domingo (University of Madrid). Hereafter, we refer to this clone as wild-type (WT). Viruses and host cells were cultured in Dulbecco’s modified Eagle’s minimum essential medium (DMEM; Gibco) with 5% heat-inactivated newborn bovine calf serum (Atlanta Biologicals) and 1% penicillin-streptomycin-L-glutamine (Gibco). Hosts for infection were baby hamster kidney (BHK-21) cells, kindly provided by E. Domingo (University of Madrid) and grown in 25-cm^2^ tissue-culture flasks at 37°C, 95% relative humidity, and 5% CO_2_ atmosphere to achieve confluent monolayers of ∼10^5^ cells/cm^2^. Low passage variants of cell lines (i.e., between 5 and 25 laboratory passages) were used for all experiments.

### Experimental Evolution of Viral Populations

Wild-type VSV was grown to high titer [≈10^8^ plaque-forming units (PFU) per mL] to create a stock lysate that was used to found five replicate populations (L1 through L5). Confluent monolayers of BHK-21 cells were infected at an initial ratio of 10 viruses per cell (*MOI* ≈ 10). This *MOI* created a high probability for multiple virus particles to co-infect individual cells, which would selectively favor spontaneously arising DIPs to gain an intracellular fitness advantage over ordinary viruses (Huang, 1973). At 24 h, an ‘undiluted passage’ was conducted: from the 3 mL supernatant containing the viral progeny, 0.12 mL was removed and used to infect a new confluent monolayer, with roughly 2.9 mL fresh DMEM. This 24-h propagation cycle was repeated 20 consecutive times for each population. After daily propagation, a sample from each population was stored at –80°C to perform subsequent plaque assays.

### Plaque Assays

A serially diluted virus sample was added to a confluent monolayer of BHK-21 cells grown in 6-well plates, and incubated for 45 min at 37°C with rocking every 15 min. Medium containing virus was removed, and replaced with a mixture of agar and 2x DMEM, and incubated for additional 24–30 h at 37°C. Cells were then fixed with 10% formaldehyde, agar and medium was removed, and cells were stained with crystal violet to visualize plaques. Titers (PFU/mL) were estimated as averages of counts from dilutions in two separate wells.

### Whole Genome Sequencing

Viral populations were sequenced at three time points in their evolution: the WT virus used to found the replicates (day 0), and the five replicate populations on days 10 and 20. Genomic RNA was extracted from 140 μL of frozen viral stock using Qiagen QiAmp mini viral spin kit. cDNA was generated by reverse transcription with Superscript II (Invitrogen), using random hexamer primers. Virus was polymerase chain reaction (PCR) amplified using GoTaq polymerase (Promega), and VSV specific primers (Supplementary Table S1). Amplicons were purified by enzymatic inactivation: Antarctic phosphatase was used to catalyze the removal of 5′ phosphate from DNA and RNA to prevent self-ligation, and Exonuclease 1 was used to catalyze the removal of nucleotides from single-stranded DNA in the 3′ to 5′ direction. Amplicons were used in Sanger sequencing at the Yale University DNA Analysis Facility on Science Hill. The coverage was between twofold and eightfold from overlapping fragments (range: 300–2100 bp in length) using multiple directional sequencing primers (Supplementary Table S1). Replicate sequences were obtained from at least two independent PCRs, and mutations called only when observed on all reads. Chromatograms were checked for quality and assembled into contigs using CLC Main Workbench 6. Whole genome sequencing constituted the consensus sequence (dominant alleles) of an evolved VSV population, regardless of the genetic background on which these alleles resided. Thus, consensus sequencing of an RNA virus population could be considered analogous to the viral quasispecies, the genetically diverse mixture of genotypes (ordinary viruses, DIPs) within the population at the time of sequencing.

### Fast Fourier Transform Analysis

Fast Fourier Transform (FFT) analysis transforms a function or set of data from the time or sample domain to the frequency domain. This means that the Fourier transform can display the frequency components within a time series of data. By inputting the virus titer data into the FFT function in MATLAB (MathWorks, Natick, MA, USA), we obtained an estimated periodicity of the oscillations (number of passages constituting a cycle) in each virus population.

### Statistical Analysis

To analyze the effect of evolved VSV population on the estimated titer of the ordinary virus subpopulation, we included the log_10_ PFU/ml data in a one-way ANOVA that included population as a model effect.

### [^3^H]Uridine Labeling

RNA synthesis was examined by direct metabolic labeling of cells 5 h post infection. Cells were exposed for 6 h to [^3^H]uridine (33 mCi/ml) in the presence of actinomycin D (10 mg/ml). Cells were harvested, and total cytoplasmic RNA was extracted and analyzed as described previously (Pattnaik et al., 1992; Wertz et al., 1994).

## Results

### Virus Population Dynamics

In serial passage, a microbial population is cultured in the laboratory for a defined period (typically 24 h) and then subsampled into fresh medium to initiate the next passage. The dilution used to obtain the subsample is termed a population ‘bottleneck’ because only a portion of the population is transferred (i.e., here, bottleneck is used differently than in population genetics, where bottlenecking indicates that an evolving population is expected to experience effects of genetic drift). To impose frequent co-infection in VSV populations we used so-called ‘undiluted’ passages, a misnomer in the scientific literature because a dilution actually occurs during this daily serial bottleneck. In our experimental evolution protocol (see the Section “Materials and Methods”) the daily dilution was 25-fold. This wide ‘undiluted’ bottleneck ensured that viruses outnumbered cells in the monolayer, promoting frequent co-infection between viruses as they replicated within host cells. By contrast, most VSV evolution studies employ much narrower population bottlenecks (typically on the order of 10^-4^–10^-6^ dilution) to specifically prevent frequent co-infection of particles in the same cell (e.g., Turner and Elena, 2000).

**FIGURE 1:**
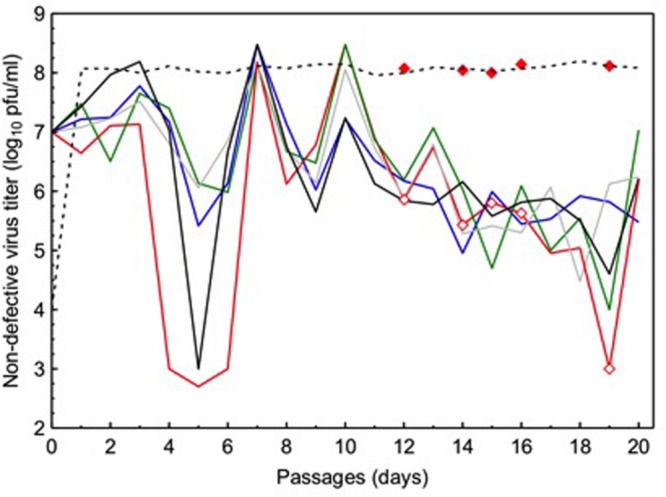
**Temporal dynamics of ordinary virus titers in five vesicular stomatitis virus (VSV) populations independently cultured for 20 passages under frequent co-infection of BHK-21 cells.** Each solid line depicts titer data from an individual population (purple line: L1; red line: L2; green line: L3; gray line: L4; black line: L5). Open diamonds indicate time points when virus samples were isolated from lineage L2, and assayed for growth on cells at low multiplicity of infection (filled diamonds). Dashed line indicates typical growth ability of a VSV lineage founded by a full-length wild-type (WT) virus genotype, and passaged in the absence of co-infection.

Because ordinary viruses have full-length genomes, their densities can be enumerated in plaque assays (see the Section “Materials and Methods”) where diluted samples of virus supernatants are plated on host monolayers to count plaques initiated by individual virus particles. In contrast, DIPs lack one or more essential virus genes and therefore cannot form plaques when infecting cells alone; thus, plaque assays can estimate ordinary virus densities, but cannot be used to measure DIP titers. At each passage in the experimental evolution, we obtained diluted samples from each evolving VSV lineage and used plaque assays to estimate the titer (PFU per mL) of ordinary viruses present in the population at each time point. These estimates of ordinary virus titers were then log_10_-transformed. Results (**Figure 1**) showed that all five experimental VSV populations (L1–L5) presented oscillating (increasing and decreasing) densities in ordinary virus titers through time. These observed population dynamics were similar across the five replicate populations, and in each case the dynamics generally resembled the oscillatory shape of a sine wave with dampened oscillations through time (see further analysis below). Some assays showed that the ordinary virus subpopulation fell to very low densities (i.e., titer ≤ 10^4^ PFU/mL; **Figure 1**); however, in all cases the ordinary viruses rebounded in number by the next time point, confirming that ordinary viruses increased in frequency (**Figure 1**). We concluded that the oscillatory dynamics were consistent with expectations from the theoretical and empirical literature on interactions between DIPs and ordinary viruses in an evolving VSV population, because undiluted passages should cause ordinary virus densities to widely fluctuate through time as these full-length viruses and DIPs experience negative frequency-dependent selection.

During the course of the experiment, all five experimental populations showed a trend of decreased titers (PFU/mL) of ordinary viruses as passage number increased. For each lineage, the average size of the full-length virus subpopulation was estimated by calculating the harmonic mean of its observed titer estimates. Hence, the estimated harmonic means equaled: L1: 4.7×10^4^ PFU/mL; L2: 3.6×10^3^ PFU/mL; L3: 1.1×10^4^ PFU/mL; L4: 6.7×10^4^ PFU/mL; L5: 1.1×10^4^ PFU/mL. These data suggested that the subpopulation sizes of the ordinary viruses were highly similar across our experiment, which was supported by results of a one-way ANOVA that showed the effect of population on ordinary virus titer was not statistically significant [*F*_(2.931,55.68)_ = 2.247; *P* = 0.094].

To further compare population dynamics across replicate lineages, we examined the log_10_ titer data of ordinary viruses using FFT analysis, which estimated the periodicity of oscillations (number of passages constituting a cycle) in each population (**Figure 1**). Results showed that the number of passages constituting a cycle differed somewhat across the five experimental populations: L1: 2.2 passages/cycle; L2: 2.3 passages/cycle; L3: 3.3 passages/cycle; L4: 3.6 passages/cycle; L5: 4.4 passages/cycle.

### Ecological Interactions Dictate Full-length Virus Productivity

The observed low productivity of ordinary viruses could be due solely to ecological interactions with DIPs, which are expected to deleteriously reduce the absolute fitness (log_10_ titer) of full-length viruses. Alternatively, the inability of ordinary viruses to achieve high titers similar to that of WT VSV (≈10^8^ PFU/mL) could have been driven wholly by evolved changes in the full-length viruses. In particular, evolution of ordinary virus resistance to DIPs could have coincided with reduced ordinary virus fecundity; this evolutionary growth trade-off would indicate inability of full-length viruses to simultaneously evolve DIP resistance while maintaining robust growth. If the absolute fitness of an evolved ordinary virus at *MOI* = 0.01 (i.e., in absence of DIPs) was similar to that of the WT ancestor grown at low *MOI* (i.e., ≈10^8^ PFU/mL), this outcome would suggest that the observed low productivity of full-length viruses on certain passage days (**Figure 1**) was due to ecological interactions with cheating DIPs. However, if the absolute fitness of an evolved ordinary virus measured at *MOI* = 0.01 was less than the ancestor (≈10^8^ PFU/mL), this result would suggest that its decreased fecundity was a trait that evolved during the course of the experiment.

To examine these competing hypotheses, we used plaque assays (see the Section “Materials and Methods”) to measure the productivity of representative ordinary viruses drawn from an evolved population, under conditions where virus particles must infect cells on their own (*MOI* = 0.01). We chose to examine ordinary virus samples isolated at five time points during the passage series belonging to population L2. In particular, we isolated samples from passages where the observed titers were relatively low (i.e., passages 12, 14, 15, 16, and 19), because these time points presumably coincided with successful cheating by DIPs that drove down the observed frequencies of ordinary viruses in the population. We used plaque purification to isolate a single clone at random from each of the five time points in population L2’s passage history. Each of these clones was used to infect BHK-21 cells at *MOI* = 0.01. After 24-h incubation, we quantified the resulting virus progeny using our standard plaque assay (see the Section “Materials and Methods”). Results (**Figure 1**) showed that all five of the ordinary virus clones had the capacity to grow to high titers (≈10^8^ PFU/mL), comparable to results for the WT ancestor grown under identical *MOI* = 0.01 conditions. The data strongly implied that the observed low titers in population L2 were not due to evolved changes in ordinary virus productivity, but were rather due to ecological interactions between the ordinary viruses and the DIPs. However, we do not dismiss the possibility that some evolutionary changes occurred in the full-length viruses in our study, and we present and discuss these data below.

### Radiolabeling Analysis Confirming DIP Presence

Although the oscillatory dynamics in **Figure 1** were consistent with expected effects of evolved DIPs on ordinary virus titers, we sought additional direct confirmation of DIP presence in the experimental virus populations. We therefore performed a preliminary analysis to visualize DIPs that were present at the endpoint (passage 20) of the study, and to estimate whether DIPs were similar across the independently passaged populations. We first obtained a sample of the supernatant from each endpoint population. These samples were then amplified at *MOI* = 10 on BHK-21 cells, and subsequently subjected to RNA extraction followed by labeling with [^3^H]uridine (see the Section “Materials and Methods”). Results (Supplementary Figure S1) showed the expected mRNA banding pattern for the five VSV genes (N, P, M, G, L; see gene description in the Section “Introduction”) when WT VSV infected BHK cells. In contrast, these results showed that DIPs were present in each endpoint population, and that these DIPs presented fewer mRNA bands than WT VSV, consistent with their relatively shortened genomes. Importantly, the observed banding patterns across the four experimental lineages that yielded data suggested that parallel DIP evolution occurred in our study; for unknown reasons one population (L2) failed to yield data in this assay, despite clearly containing viable viruses at passage 20 (**Figure 1**). These preliminary data suggested that the independently passaged VSV lineages had converged in terms of the majority DIPs present at passage 20, at least in the four populations that yielded results.

### Genomic Analysis of Evolved Populations

Sanger sequencing can be used to generate a consensus sequence for an evolved virus population, which usefully indicates the alleles that distinguish the evolved population from its ancestor. Although Sanger sequencing cannot determine whether observed mutations exist on a common genetic background (i.e., it cannot identify unique haplotypes), the method can be used to efficiently compare across replicate populations to test whether treatment conditions tended to select for parallel mutations across independently evolved lineages (e.g., Remold et al., 2008; Alto et al., 2013). We employed this approach to test whether parallel changes occurred across the five experimental populations, and whether certain mutations coincided with the early phase of the experiment typified by high amplitude oscillations versus with the later phase exemplified by dampened oscillations. Thus, the genomic analysis further tested for repeatability across the evolved populations, and also tested whether the observed changes in oscillatory dynamics coincided with differing molecular substitutions in the early versus later phases of the study.

Because VSV is a single-stranded RNA virus with a typically high mutational error rate, we expected that 20 passages of experimental evolution would be sufficient to detect polymorphic loci and/or fixed substitutions that separated the evolved lineages from their common ancestor virus, and possibly from one another. Importantly, this expectation assumes that the high *MOI* environment – and presumably DIP presence – exerts strong enough selection for the VSV consensus genome sequence to change in the evolutionary time allowed, especially due to beneficial mutations that spontaneously appear and spread to fixation. Twenty passages in a novel environment, such as a new tissue-culture host or incubation temperature, are sufficient for the VSV consensus sequence to change via fixation of adaptive mutations (e.g., Remold et al., 2008; Alto et al., 2013). In contrast, if VSV is passaged on the typical BHK-21 lab host at 37°C, 20 passages do not typically lead to many detectable changes because the virus is already relatively fit in this environment (e.g., Alto et al., 2013). Finally, we noted that the highly variable population sizes through passage time in our study (**Figure 1**) could prevent natural selection from operating efficiently, thus potentially allowing genetic changes to accrue in ordinary virus and/or DIP subpopulations through genetic drift. In summary, we expected that both adaptive and non-adaptive mutations could increase in frequency during the experiment, making these alleles detectable through consensus population sequencing.

To examine the occurrence of molecular evolution and evidence for parallelism in our study, we focused our attention on the two phases of the experiment that clearly differed in the observed oscillatory dynamics within the evolving lineages: days 0 to 10, and days 10–20 (**Figure 1**). In particular, we defined the ordinary virus-population dynamics occurring between days 0 and 10 as the ‘early phase,’ where oscillations showed relatively greater amplitude than between days 10 and 20, defined as the ‘late phase.’ Foremost, we compared the consensus sequence of each lineage across these two phases to test whether (i) observed molecular changes separated the evolved lineage from the common ancestor, indicating that molecular evolution via selection and/or drift occurred in the experiment, and (ii) whether different alleles dominated the population in the early versus late phase. In theory, the differing oscillatory dynamics in the early and late phases could be entirely driven by ecological interactions between full-length and DIP viruses that appeared in the early phase. If true, we would expect that a lineage’s consensus sequence at day 10 would be highly similar (or even identical) to its sequence at day 20, perhaps with some early-phase polymorphic loci transitioning to fixed substitutions in the later phase. Alternatively, we might observe highly different consensus sequences in the early versus late phases, suggesting that the differing oscillatory dynamics were driven (at least in part) by both ecological interactions as well as evolutionary interactions, such as extended co-evolutionary changes between ordinary virus and DIP genotypes. Last, overall comparisons in molecular data among lineages (i.e., similarities in consensus sequences) were used to further gage the degree of repeatability in our study, especially occurrence of parallel evolution.

**FIGURE 2:**
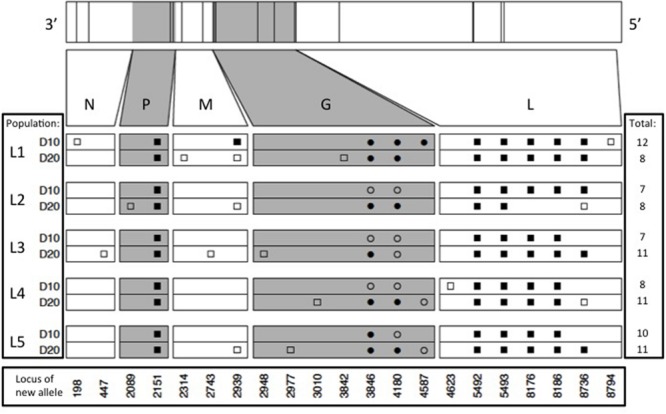
**Locations of new mutations relative to the ancestral sequence in the evolved VSV populations, at days 10 and 20 of experimental evolution.** Ticks on bars across top indicate physical locations of mutations in the genome. Viral nucleoprotein (N), phosphoprotein (P), matrix protein (M), glycoprotein (G), and large polymerase protein (L) genes are indicated on both scaled and unscaled bars. Within the 10 lower bars, each symbol represents a new nucleotide. Genome location is shown at bottom. Populations sharing symbols in the same locus share the new allele. Squares and circles represent synonymous and non-synonymous changes, respectively, and filled and open symbols represent fixed and polymorphic changes, respectively.

Results showed that the founding VSV ancestor in our study contained 102 mutations separating it from the canonical annotated VSV Mudd-Summers strain Indiana serotype (GenBank #NC_001560.1). In itself, this result is not overly surprising because the two strains have been used in separate laboratories for decades, under unknown but presumably non-identical culture conditions. More importantly for the current study, we identified a total of 93 allele substitutions and/or polymorphisms that separated the consensus sequences of the five evolved lineages from their common ancestor. These data are summarized in **Figure 2**, which shows the locations of fixed, polymorphic, synonymous, and non-synonymous changes, and indicates whether the mutation is observed by day 10 (early phase) versus appears between days 10 and 20 (late phase). In general, most of the observed mutations occurred in the early phase of the study; across all five lineages, 42 mutations were detected by day 10 and only 14 additional mutations separated the day 20 consensus sequences from their day 10 counterparts (**Figure 2**). Moreover, the vast majority of these additional mutations (11 of 14 changes) were polymorphic at day 20. Overall, these comparisons indicated that most of the molecular evolution in our study had occurred in the early phase when oscillatory dynamics showed relatively high amplitudes (cf. **Figures 1** and **2**).

Our molecular data also indicated that abundant parallel evolution occurred across the five lineages (**Figure 2**). Because VSV is an ssRNA virus, we note that synonymous mutations can affect the secondary structure of the virus and thus potentially impact fitness. One fixed, synonymous mutation occurred in the gene for the VSV phosphoprotein in all five lineages by day 10 (nucleotide position 2151), and was still present at day 20. In the VSV glycoprotein gene, all five lineages showed a non-synonymous mutation at position 3846 (D→N) and at locus 4180 (T→K); one or both of these mutations had either fixed early in an evolved lineage (e.g., L1, L5), late in the experiment (e.g., L2, L4), or remained polymorphic throughout the study. The Large protein showed the largest number of parallel substitutions across the five lineages (**Figure 2**). Here, all lineages showed the same five mutations (changes at loci 5492, 5493, 8176, 8186, and 8736), and most of these substitutions fixed by day 10 (early phase). Interestingly, lineage L2 fixed two of these mutations by day 10, but then reverted back to the ancestral sequence by day 20.

Aside from the large number of parallel mutations across lineages, we observed nucleotide changes specific to a single lineage, or shared among a subset of the replicate lineages (**Figure 2**). However, these changes tended not to bridge the early and late phases of the experiment, and were often observed to be polymorphic. For these reasons, the minority non-parallel substitutions in our study may be considered less impactful, but this tentative conclusion warrants further investigation.

## Discussion

Our study examined the repeatability of population dynamics and molecular substitutions across five independently passaged VSV lineages, cultured under frequent virus co-infection that selects for evolution of DIPs (virus cheaters). After 20 days of serial passage that facilitated negative frequency-dependent selection between ordinary viruses and DIPs, all five experimental VSV populations displayed a cycling pattern consistent with theoretically predicted effects of DIPs on full-length virus densities (Palma and Huang, 1974; Depolo et al., 1987; Kirkwood and Bangham, 1994). A molecular assay confirmed that DIPs were present in the endpoint virus populations, and growth of representative full-length viruses at low *MOI* (absence of DIPs) showed that these strains retained robust reproductive ability. Changes in oscillatory dynamics (early versus late phases of the experiment) and patterns of molecular substitutions were highly similar across the five VSV lineages. Altogether, these results suggested that ecological interactions between ordinary viruses and cheaters were extremely repeatable across independently passaged lineages, and that any contribution of virus evolutionary changes to the observed dynamics within lineages was dictated by molecular substitutions occurring relatively early in virus evolution.

Our study was motivated by efforts to better understand fundamental antagonistic virus–virus ecological interactions (Wensel et al., 2003). Only a tiny minority of virus species function in human disease, but the medical importance of viral pathogens causes them to receive undue attention, and defective RNA viruses are sometimes implicated as important (e.g., Gan et al., 2002; Aaskov et al., 2006; Kim et al., 2007). One example is hepatitis delta virus, a medically relevant defective virus often associated with hepatitis B virus in severe cases of chronic active hepatitis (Taylor, 1999). In addition, clinical samples taken from humans suffering from dengue fever show that some mosquito-transmitted dengue virus populations are dominated by defective variants, at least in certain geographical regions (Aaskov et al., 2006). However, we note that DIPs and other defective viruses are necessarily difficult to detect because these variants do not form plaques in standard assays. Thus, the ecological and evolutionary importance of defective viruses in disease systems may be grossly underestimated. For this reason, experiments on the evolutionary ecology of DIPs and other defective viruses remain fertile areas for research. Our work hints that certain aspects of these empirical studies may be more tractable than expected, because virus–virus interactions can be highly conserved within a particular system; of course this assumption must be verified by extending such work beyond the VSV system presented here.

Previous empirical (von Magnus, 1951; Lief and Henle, 1956; Palma and Huang, 1974; Horiuchi, 1983; Depolo et al., 1987) and theoretical (Kirkwood and Bangham, 1994) data predicted the oscillating community dynamics between ordinary and DIP viruses observed in the current study. Such oscillations are generally not observed when VSV is evolved under identical culture conditions and similar time periods as our study, but at strictly low *MOI* conditions that do not foster selection for cheater viruses (e.g., Turner and Elena, 2000). Our FFT analysis confirmed the cyclical nature of these dynamics, but suggested that cycle periodicity differed across the experimental populations, ranging between 2.2 to 4.4 passage days per cycle. It is unknown whether some divergence between the populations helps to explain these observations; e.g., due to differing ‘cheating abilities’ among DIPs evolved in separate populations. Alternatively, these cycling differences may be purely stochastic, such as due to subtle differences across the populations in the state of host cells on certain passage days (e.g., random differences in host cell physiology or chance fluctuations in available host numbers) and/or due to non-parallel genetic differences among lineages. We note that the FFT analysis is usually used for larger data sets, and that the accuracy of our cycling period estimates may therefore be poor (i.e., weak resolution of cycle measurements). Despite the estimated difference in cycle numbers across test populations, the overall cycling patterns (especially dampened oscillations through time) were remarkably consistent and repeatable.

Although consensus sequencing offers a convenient ‘snapshot’ of the allele diversity in a virus lineage, these gross changes necessarily omit key details regarding the genetic backgrounds on which mutations arise and accumulate. Nevertheless, our comparison of consensus sequences in the early versus late phases of the experiment, both within and across populations, strongly suggested that molecular evolution occurred and that it was highly repeatable. We concluded that effects of genetic drift did not generally overwhelm those of natural selection, because abundant parallel substitutions were observed and these could not have occurred by chance (Remold et al., 2008). Thus, the available evidence indicated that the most obvious dynamic changes (dampened oscillations that characterized early versus late phases) were probably driven by similar changes across the five lineages, which occurred relatively early during the study. Apparently, frequent virus co-infection exerted strong selection for particular synonymous and non-synonymous substitutions early in the experiment, creating parallel consensus genetic changes across the five virus ‘communities’ (full-length and DIP genotypes). This parallelism mirrored highly similar population dynamics, especially approaches to roughly the same ecological equilibrium across the five lineages. Additional work beyond the current study, especially VSV reverse genetics, would be necessary to distinguish which of the parallel changes truly constituted adaptive mutations fixed by selection, versus those that rose to high frequencies via other processes (e.g., via genetic hitchhiking).

The population dynamics observed in our experimental populations were reminiscent of those observed in macro-organism predator–prey and host–parasite systems, because predator/prey and parasite/host relative densities can oscillate in a similar fashion (Begon et al., 1996; Gotelli, 1998). In these classic macro-organism examples predators and parasites can sometimes regulate prey and host numbers. For example, removing or minimizing the predator allows the prey to achieve a population size that is much larger, because they are no longer effectively regulated. By analogy, in our system DIPs can be considered the “predator,” and ordinary viruses the “prey.” By growing ordinary viruses on their own, we determined that presence of DIPs caused the full-length viruses to be regulated at a population size much lower than they could ordinarily achieve. This result suggested that the dampened oscillatory dynamics were driven more by ecological than evolutionary interactions, because representative full-length viruses from lineage L2 had not changed in their ability to produce large population sizes when challenged to grow at low *MOI* on the BHK host cells. Previous work suggested that ordinary viruses could evolve specific resistance to a particular DIP (e.g., Roux et al., 1991), and the possibility existed that coevolution with DIPs could select for resistance mechanisms that were costly for ordinary virus fitness in our study. Rather, we observed that full-length viruses maintained robust growth despite selection favoring DIPs. Moreover, this apparent avoidance of a possible evolutionary growth trade-off indicates that the ordinary viruses should maintain the potential for evolved improvement if they were released from ecological interactions with DIPs. However, further work could examine the intriguing possibility that ordinary viruses suffer evolutionary contingency, if placed in a novel selective environment, such as a new host or incubation temperature. That is, following their ecological history of exposure to cheating by DIPs and any associated genetic-architecture changes in the full-length virus genome, negative epistasis may limit the evolvability of these ordinary viruses compared to otherwise identical viruses cultured for the same period of time in absence of cheating. Other studies show that various ecological circumstances can prove consequential for performance and evolvability (potential to adapt) of RNA viruses in novel environments, because the selective environment causes changes to viral genetic architecture (Turner et al., 2010, 2012; Alto et al., 2013; Goldhill, 2015). Thus, an intriguing possibility for future work would be to challenge the endpoint full-length virus populations to grow or evolve under new circumstances, to reveal whether historical exposure to cheating affects their performance and/or potential to further adapt.

## Author Contributions

EW: main execution of genome sequencing and phenotypic analysis of evolved virus populations; writing and editing of manuscript. NM: design and execution of evolution experiment; writing and editing of manuscript. BW: sequence and other data analysis; editing and commenting on manuscript. VB: execution of experiments confirming presence of DIPs. SW: design of experiments confirming presence of DIPs; feedback on manuscript. PT: design and interpretation of experiments; writing and editing of manuscript.

## Conflict of Interest Statement

The authors declare that the research was conducted in the absence of any commercial or financial relationships that could be construed as a potential conflict of interest.
