# Supplementary material for: Repeatable Population Dynamics among Vesicular Stomatitis Virus Lineages Evolved under High Co-infection
Source: Front Microbiol. 2016 Mar 31;7:370. doi: 10.3389/fmicb.2016.00370 (PMC4815288; doi:10.3389/fmicb.2016.00370)
Supplement: Supplementary file 1 [file Table_1.DOCX]

Table S1. Primers used for PCR amplification and sequencing of VSV samples.

| **Primer Name** | **Sequence (5' to3')** |
| --- | --- |
| 18F | ACGAAGACAAACAAACCA |
| 27F | CGAAGACAAACAAACCATTATTATC |
| 676F | TGTGGGGAAATGACAGTAATT |
| 806R | GTGTCCAAATGTTTGCCAATG |
| 987F | AATTGACAGCTCTTCTGCTC |
| 1033F | ATTGACAGCTCTTCTGCTCA |
| 1043R | ACAAACCTGCTGTAGTAA |
| 1259R | TTGCAGTGACATGACTGCTC |
| 1356F | GTCAGAGTTTGACAAATGACCC |
| 1574R | TGGTTCAGATTCTGTGTCAG |
| 1770F | AAAGACCTTACGGTTGACA |
| 1838R | CAGATTCCAGTATTTGGCAC |
| 2253F | CCAATCCATTCATCATGA |
| 2280R | TTTCTTACCTTTCCCCTT |
| 2502F | TCAGAACATACTCAGATGTGG |
| 2551R | TTTTGTAGAAGGGACGTT |
| 2844R | CTTCTCTCTGAAATCAGA |
| 3063R | CATAGTGTCAAGGAAACA |
| 3189F | GAAAAATGTTCCTTCCAATTACCA |
| 3312R | ACAAGTAGTGACCCATTT |
| 3663R | GTCCATGGAAATGAGGTT |
| 3835F | AGACTCCCATCAGGTGTCTGGTT |
| 3922R | TTAGACTTACATCCACTG |
| 4332F | TCATCTTAGCTCAAAGGCTC |
| 4621R | TGAAGAATCTGTTGTGCAGG |
| 4968R | GGGATTGGCTTGACATGATG |
| 5196F | GACTCATTCAAAATTCTCGC |
| 5250R | CAATTCCACCTCAGAGACAG |
| 5489R | TGCTCTGAGAACAGGTTGTC |
| 6071F | TGGAGATAAATGGCATGAAC |
| 6096R | TGGGTCTAGTAAGTCGGGTA |
| 6354R | TTTCCAAGACATTAGGGAGA |
| 6675R | GTCTGGTCTTCCATTGTAGT |
| 6957F | GCTCTCAATCAAATGGTTTC |
| 6993R | AAGTCCTAACTTCCCTGTCC |
| 7251R | CAAGAGTCTAGCAAATGTCC |
| 7252F | TTGGGACATTTGCTAGACTC |
| 7252R | TTGGGACATTTGCTAGACTC |
| 7598R | GCTGGACTCATTCCCATAGC |
| 7859F | TCGTACTATTCGGAACTCCT |
| 7886R | GTCCTCACAATCAAATCATC |
| 8150R | CGTGAACTAAAGACGTCA |
| 8478R | GTCTGTAGTTGCCATCAACC |
| 8756F | GTGGGGACAAGAGATAAAAC |
| 9062R | AGGAATGGAGGTGATACACT |
| 9344F | GCCATCTTTATCTGGGAAAGA |
| 9395R | CACCCCTCTCCTGATCTTAG |
| 9626F | AGATGCCTCCAAGAATCC |
| 9666R | TATAATGAGCGCCAGTTG |
| 10173R | GTCGACCGTCTTGAACATGG |
| 10337F | TACGCATTCCAGTCATCAG |
| 10389R | AATGAATTGGGAGGGAATC |
| 10587R | ATCAGAGTAGGACCGATACC |
| 10699R | GAATGATTGCTGGATAACTGC |
| 11143R | ACGAAGACCACAAAACCAG |
