# Supplementary material for: Repeatable Population Dynamics among Vesicular Stomatitis Virus Lineages Evolved under High Co-infection
Source: Front Microbiol. 2016 Mar 31;7:370. doi: 10.3389/fmicb.2016.00370 (PMC4815288; doi:10.3389/fmicb.2016.00370)
Supplement: Supplementary file 2 [file Image_1.PDF]

Supplementary Material

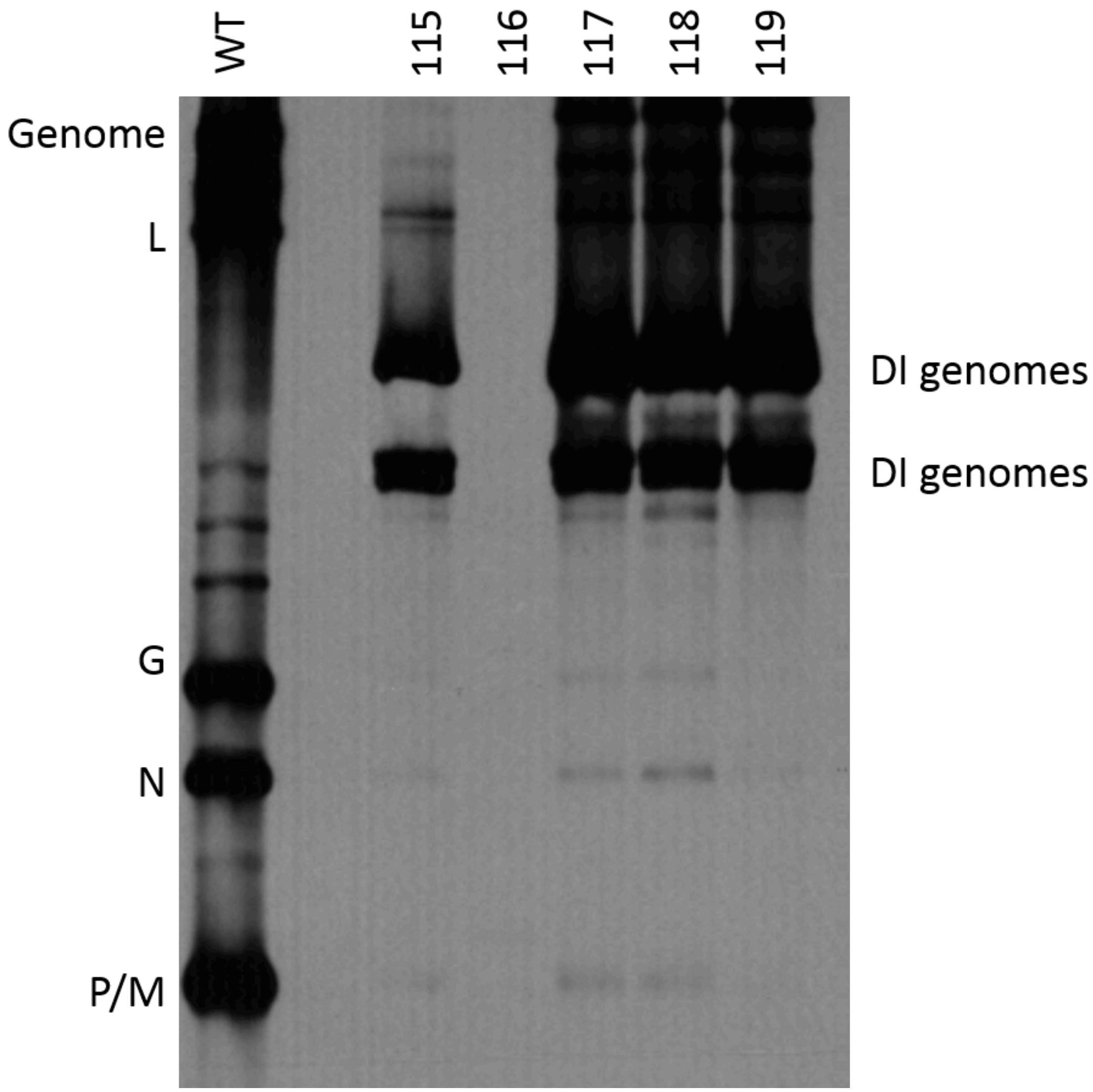

Fig. S1. The [^{3}H]uridine assay showed that BHK cells infected by wildtype (WT) VSV show the full set of expected mRNAs in VSV infected cells. By contrast, a very different banding pattern was observed in assays where cells were infected by passage-20 samples from the evolved VSV populations (115: L1; 116: L2; 117: L3; 118: L4; 119: L5; note that the L2 population sample failed to amplify for unknown reasons). Evolved populations showed only very low levels of the mRNA but highly prominent different bands that represent the DIP genomes. In addition, the similar banding patterns across the 4 evolved populations suggested that parallel evolution of DIPs occurred in the independently evolved lineages. The exposure of the gel selected here is intended to allow visualization of the low levels of the mRNA made by the virus in the presence of the highly abundant DIP genomes. This acid-agarose gel separates RNA on the basis of size and charge and therefore using size markers is not appropriate. mRNA bands pertaining to each of the 5 VSV genes (L, G, N, P and M) are noted in the figure.
